# Applying self-powered sensor and support vector machine in load energy consumption modeling and prediction of relational database

**DOI:** 10.1038/s41598-023-46414-3

**Published:** 2023-11-04

**Authors:** Dexian Yang, Jiong Yu, Zhenzhen He, Ping Li, Xusheng Du

**Affiliations:** School of Information Science and Engineering, Xinjiang University, Ürümqi, 830046 China

**Keywords:** Information technology, Scientific data

## Abstract

This study explores the analysis and modeling of energy consumption in the context of database workloads, aiming to develop an eco-friendly database management system (DBMS). It leverages vibration energy harvesting systems with self-sustaining wireless vibration sensors (WVSs) in combination with the least square support vector machine algorithm to establish an energy consumption model (ECM) for relational database workloads. Through experiments, the performance of self-sustaining WVS in providing power is validated, and the accuracy of the proposed ECM during the execution of Structured Query Language (SQL) statements is evaluated. The findings demonstrate that this approach can reliably predict the energy consumption of database workloads, with a maximum prediction error rate of 10% during SQL statement execution. Furthermore, the ECM developed for relational databases closely approximates actual energy consumption for query operations, with errors ranging from 1 to 4%. In most cases, the predictions are conservative, falling below the actual values. This finding underscores the high predictive accuracy of the ECM in anticipating relational database workloads and their associated energy consumption. Additionally, this paper delves into prediction accuracy under different types of operations and reveals that ECM excels in single-block read operations, outperforming multi-block read operations. ECM exhibits substantial accuracy in predicting energy consumption for SQL statements in sequential and random read modes, especially in specialized database management system environments, where the error rate for the sequential read model is lower. In comparison to alternative models, the proposed ECM offers superior precision. Furthermore, a noticeable correlation between model error and the volume of data processed by SQL statements is observed. In summary, the relational database ECM introduced in this paper provides accurate predictions of workload and database energy consumption, offering a theoretical foundation and practical guidance for the development of eco-friendly DBMS.

## Introduction

Data volume and generation speed have surged with the rapid growth and widespread adoption of mobile internet, the Internet of Things, and cloud computing technologies. Analyzing and modeling energy consumption under varying workloads is essential for creating energy-efficient and eco-friendly database management systems (DBMSs)^1^. However, conventional mechanical wireless vibration sensors (WVSs) are unsuitable for specific environments, and the limitations in power supply have restricted the advancement of wireless sensor networks (WSNs). In the face of energy-intensive big data environments, exploring energy consumption models (ECMs) for relational database workloads, referred to as the ECM, has emerged as a pivotal pursuit for crafting energy-efficient DBMSs^2^. This research direction encounters multiple hurdles and challenges. Firstly, traditional WVSs fall short in meeting the demands of specialized environments and fail to capture real-time and precise vibration energy data. Secondly, the constrained power supply remains a hindrance for WSNs, limiting their application scope and performance. Furthermore, due to the intricacy and high energy consumption traits of big data environments, the precise analysis and modeling of energy consumption within relational database workloads, along with the ability to make predictions and optimizations, present formidable challenges. In response to the aforementioned issues, this paper seeks to offer a solution by employing the least square support vector machine (LSSVM) algorithm and self-powered WVSs to construct a model for energy consumption in relational database workloads. The introduction of self-powered WVSs tackles traditional sensors’ limitations in specific environments, while the LSSVM algorithm equips the model with effective energy consumption prediction capabilities. This model can achieve a more precise analysis and assessment of energy consumption within relational database workloads, thereby providing a theoretical foundation and practical guidance for creating energy-efficient DBMS. Hence, the significance of this paper lies in its ability to overcome the limitations of applying WVSs in specialized settings and combining the LSSVM algorithm to establish a model for energy consumption in relational database workloads tailored to big data environments, thereby offering a novel solution for constructing energy-efficient DBMS.

Priestly et al.^3^ introduced an innovative hybrid optimization algorithm to forecast monthly precipitation and empirically verified its effectiveness and superiority. This hybrid model leverages the strengths of swarm intelligence optimization algorithms and ensemble learning algorithms, improving the identification of features and model parameters for enhanced prediction accuracy and reliability^3^. In recent studies, Hu et al.^4^ presented an energy-saving approach that combined hardware and software to create and validate the ECM for each component of a system, with the relational database’s energy consumption as the key evaluation metric. Dembele et al.^5^ estimated energy consumption through Greenplum database queries, investigated strategies to enhance connection energy efficiency and evaluated the variance in connection calculation time under different scales and connection modes. Zhou et al.^6^ focused on the calculation and energy-efficient collaborative scheduling for Green DBMS centers to optimize their overall energy efficiency. In a study by Asha and Santhosh^7^, an energy-balanced routing protocol tailored for WSNs was developed to mitigate monitoring blind spots arising from uneven energy consumption distribution among nodes. Existing research predominantly revolves around predicting database energy consumption, with less emphasis on gathering sensor energy data and predicting load energy consumption within relational databases^8^. The WVS network comprises various nodes, often located in hard-to-reach positions and immobile. Power sources limit the expansion of the WVS network, mandating nodes to harvest energy from their surroundings, achieve self-sufficiency, and manage equipment status monitoring^9^. Self-powered electrical WVSs harness mechanical vibration signals and energy attributes to monitor mechanical equipment operations^10^. These self-powered WVS nodes capture energy generated during mechanical equipment operation for subsequent utilization^11^. On a different note, the LSSVM is an effective tool for data analysis. It shares the attributes of support vector machine (SVM), which include suitability for small sample sizes, handling nonlinearity, high dimensionality, and superior prediction accuracy. SVM is widely adopted for tasks like power load forecasting and constructing ECMs^12^. In summary, current research on analyzing and modeling energy consumption in relational databases has some shortcomings and deficiencies. Firstly, traditional mechanical WVSs are unsuitable for specialized environments, preventing the collection of real-time and accurate vibration energy data. Secondly, the limitation of convenient power supply hinders the progress of WSNs, restricting their scope and performance. Moreover, in the complex and high-energy big data environments, accurately analyzing, modeling, predicting, and optimizing the energy consumption of relational database workloads poses a challenging problem. The solution proposed in this paper addresses these limitations by introducing self-powered WVSs and utilizing the LSSVM algorithm, providing effective energy consumption prediction capabilities. Furthermore, the model presented in this paper offers significant advantages in analyzing and modeling energy consumption in relational database workloads. In comparison to models such as a LSSVM with Improved Multi-Verse Optimization, Long Short-Term Memory with Information Gain, Relevance Vector Machine with Improved Multiple Regression Fitness Optimization, Extreme Learning Machine with Joint Feature Optimization, and Support Vector Machine with Fast and Flexible Adaptive Particle Swarm Optimization, the model proposed here overcomes the limitations of traditional sensors and provides more accurate energy consumption prediction capabilities. Furthermore, this model specifically focuses on energy consumption in relational database workloads, offering a crucial theoretical foundation and practical guidance for the development of energy-efficient green DBMSs. In conclusion, the model presented here demonstrates outstanding advantages in energy analysis and modeling.

Building upon the theoretical foundations discussed earlier, this study applies the principles of energy harvesting from self-powered WVSs and the ECM based on an LSSVM to predict the energy consumption of relational database workloads. The accuracy of the proposed ECM is validated through comparisons with other methods. The most innovative aspect is the integration of the energy harvesting principles of self-powered WVSs with LSSVM, effectively managing the energy consumption of relational databases. This research strongly emphasizes energy consumption issues in the DBMS design process, aiming to explore sustainable, low-energy, green DBMSs. This study is expected to provide valuable theoretical references to enhance the stability and accuracy of dynamic ECM in various system environments. This integrated approach and research direction bring significant advantages and innovation to the field of database energy consumption, offering fresh insights for the development of energy-efficient and optimized DBMSs.

## Theoretical basis and research methods

### Theory of self-powered electric WVS

#### Node structure of self-powered WVS network

**Figure 1:**
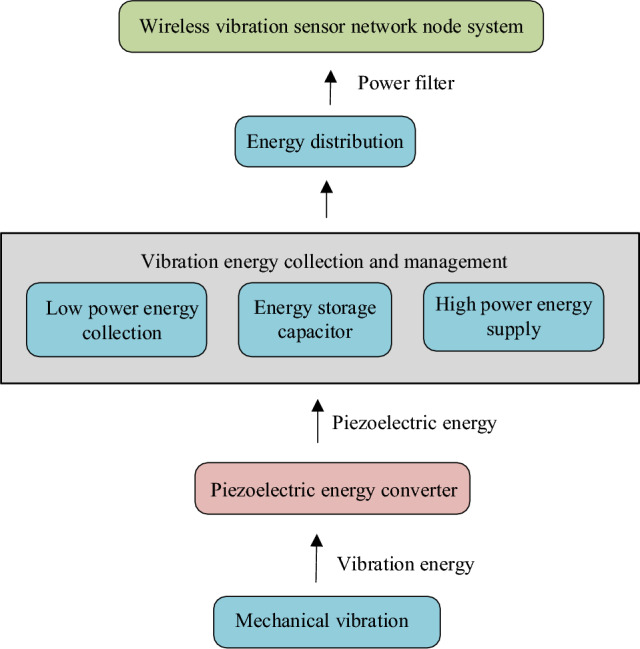
Comprehensive structure of self-powered WVS network nodes.

The self-powered WVS network comprises numerous nodes, typically positioned in concealed locations. These self-powered WVS nodes harness mechanical vibration signals and energy properties to oversee the status of mechanical equipment^13^. They operate autonomously, and the comprehensive network architecture is depicted in Fig. 1.

Figure 1 illustrates the network node’s components, including the piezoelectric transducer (PET), a vibration energy collection and management system, and the WVS node^14^. The PET operates based on the piezoelectric effect, generating alternating energy. Subsequently, the vibration energy collection and management system efficiently gathers and handles the PET’s low-power energy. This system transforms the weak energy into low-power energy for collecting high-power energy for supply and manages energy distribution through a power filter. This collected energy is then utilized to power the self-powered WVS network nodes^15^.

#### Structure of vibration energy collection and management system

**Figure 2:**
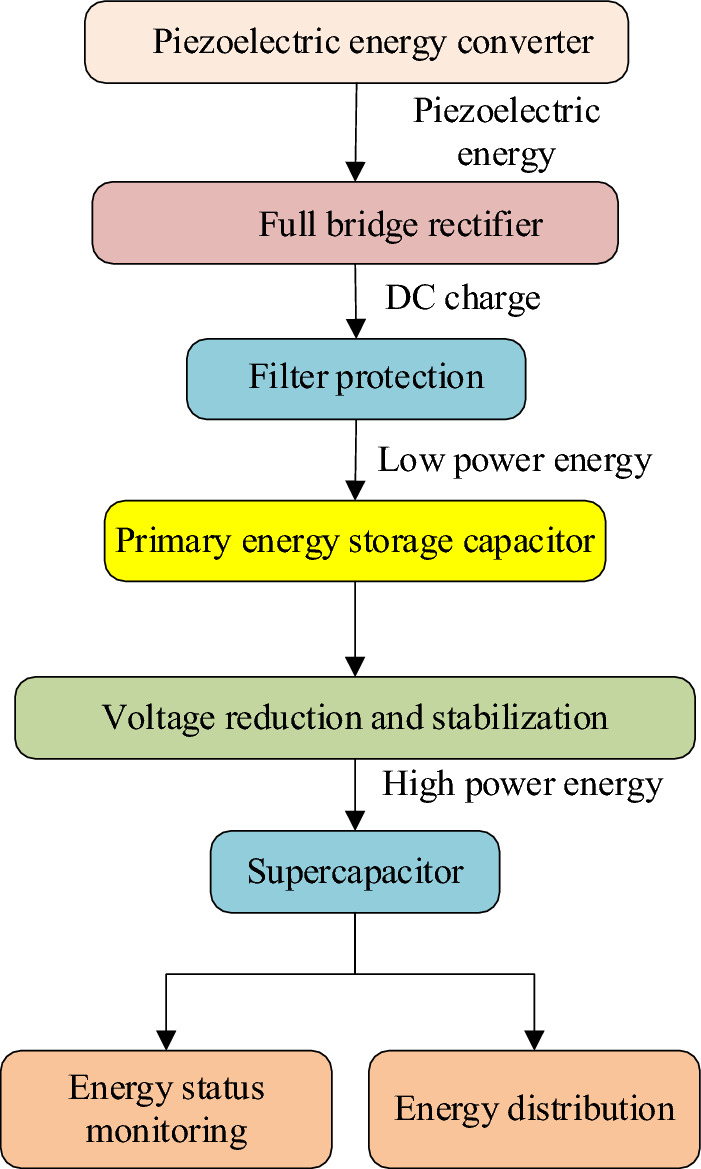
Design of the vibration energy collection and management system.

The vibration energy collection and management system serves as a vital energy source for self-powered WVS network nodes. It harnesses low-power energy generated by PET and transforms it into an abundant energy supply to support the high-power operations of these nodes^16^. Figure 2 illustrates the system’s configuration.

The vibration energy collection and management system comprises several key elements: a voltage energy converter, full bridge rectifier, filter protection equipment, energy storage capacitor, step-down and voltage stabilization equipment, low-power energy collection equipment, high-power energy supply equipment, supercapacitor, and energy status monitoring. This system is responsible for converting the mechanical vibration-induced low-power energy into high-power energy^17^.

### LSSVM prediction algorithm

#### LSSVM theory

In contrast to neural networks, SVMs are well-suited for handling small datasets while also accommodating nonlinearity, high-dimensionality, and ensuring accurate predictions, particularly when dealing with limited training samples^18^.

**Figure 3:**
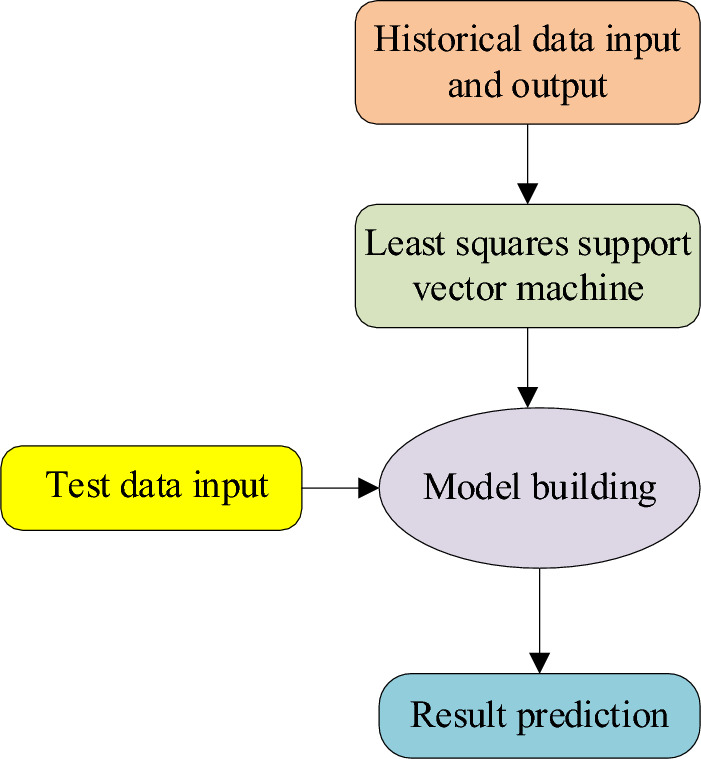
Framework of the LSSVM algorithm.

The LSSVM, a variant of the least squares regression model, proves highly effective for data analysis^19^. The algorithm’s framework is visually outlined in Fig. 3.

Figure 3 illustrates the modeling process comprising two essential steps: training and testing. During the training phase, the model is constructed using historical data, taking data inputs and generating corresponding outputs. The model is fed with new, unknown data in the testing phase to produce predictive outcomes^20^.

#### LSSVM modeling

Consider a training sample denoted as $A=\left({X}_{i}, {Y}_{i}\right)$, where $i=\mathrm{1,2},\dots ,n$. Here, ${X}_{i}\in {R}^{m}$ represents the input with *m* dimensions, ${Y}_{i}\in R$ represents the one-dimensional input, and *n* denotes the number of training samples. The transformation takes the original sample space of the prediction data from a nonlinear estimation problem to a linear function estimation problem in a high-dimensional space. This transformation can be mathematically expressed as Eq. (1).

1 $$f\left(X\right)={w}^{T}\phi \left(X\right)+B$$

In Eq. (1), $w={\left[{w}_{1},{w}_{2},\dots ,{w}_{n}\right]}^{T}$ represents the vector of weight coefficients; $\phi (X)={\left[{\phi }_{1}(X),\dots ,{\phi }_{n}(X)\right]}^{T}$ is the mapping function for the kernel space; $B$ stands for the bias term.

Following the principle of structural risk minimization, the regression problem formulates LSSVM as an optimization problem with equality constraints^21^, as shown in Eq. (2).

2 $$ \left\{ {\begin{array}{*{20}l} {\mathop {min}\limits_{w,e} J\left( {w,e} \right) = \frac{1}{2}w^{{\text{T}}} w + \gamma \mathop \sum \limits_{i = 1}^{n} e_{i}^{2} } \\ {{\text{s}}.{\text{t}}{.}\;Y_{{i_{i} }} = w^{{\text{T}}} \phi \left( {X_{i} } \right) + B + e_{i} ,\;i = 1, \ldots ,n} \\ \end{array} } \right. $$

In Eq. (2), $w$ is the weight coefficient; $e$ represents the vector composed of introduced relaxation factors; ${e}_{i}$ denotes the $i$th relaxation variable; $\gamma$ stands for the regularization constant.

The Lagrange function can be constructed by introducing the Lagrange multiplier vector $\xi$, as shown in Eq. (3).

3 $$ L\left( {w,B,e,\xi } \right) = J\left( {w,B,e} \right) - \mathop \sum \limits_{i}^{N} \xi_{i} \left[ {w^{T} \varphi \left( {X_{i} } \right) + B + e_{i} - Y_{i} } \right] $$

In Eq. (3), ${\xi }_{i}$ is the Lagrange multiplier.

Calculating the partial solution of Eq. (3) yields the optimal solution condition, as expressed in Eq. (4).

4 $$ \left\{ {\begin{array}{*{20}l} {\frac{\partial L}{{\partial w}} = 0 \to w = \mathop \sum \limits_{i = 1}^{N} \xi_{i} \varphi \left( {X_{i} } \right)} \\ {\frac{\partial L}{{\partial B}} = 0 \to \mathop \sum \limits_{i = 1}^{N} \xi_{i} = 0} \\ {\frac{\partial L}{{\partial e_{i} }} = 0 \to \xi_{i} = \gamma e_{i} } \\ {\frac{\partial L}{{\partial \xi_{i} }} = 0 \to w^{T} \varphi \left( {X_{i} } \right) + B + e_{i} - Y_{i} = 0} \\ \end{array} } \right. $$

Transforming Eq. (4) into a set of linear equations leads to Eq. (5).

5 $$ \left[ {\begin{array}{*{20}l} 0 & 1 & \cdots & 1 \\ 1 & {K\left( {X_{1} ,X_{1} } \right) + \frac{1}{\gamma }} & \cdots & {K\left( {X_{1} ,X_{n} } \right)} \\ \vdots & \vdots & \ddots & \vdots \\ 1 & {K\left( {X_{n} ,X_{1} } \right)} & \cdots & {K\left( {X_{n} ,X_{n} } \right) + \frac{1}{\gamma }} \\ \end{array} } \right]\left[ {\begin{array}{*{20}l} B \\ {\xi_{1} } \\ \vdots \\ {\xi_{n} } \\ \end{array} } \right] = \left[ {\begin{array}{*{20}l} 0 \\ {Y_{1} } \\ \vdots \\ {Y_{n} } \\ \end{array} } \right] $$

Equation (5) can be simplified as shown in Eq. (6).

6 $$ \left[ {\begin{array}{*{20}l} 0 & {1_{n}^{T} } \\ {1_{n} } & {{\Omega } + \frac{1}{\gamma }E_{n} } \\ \end{array} } \right]\left[ {\begin{array}{*{20}l} B \\ \xi \\ \end{array} } \right] = \left[ {\begin{array}{*{20}l} 0 \\ Y \\ \end{array} } \right] $$

In Eq. (6), ${E}_{n}$ denotes the *n*-order identity matrix, and $Y={\left[{Y}_{1},\dots ,{Y}_{n}\right]}^{T}$ denotes the output of training data.

The prediction model coefficients $B$ and $\xi$ are calculated by Eqs. (7) and (8).

7 $$B=\frac{{1}_{n}^{T}{\left(\Omega +\frac{1}{\gamma }{E}_{n}\right)}^{-1}Y}{{1}_{n}^{T}{\left(\Omega +\frac{1}{\gamma }{E}_{n}\right)}^{-1}{1}_{n}}$$

8 $$ \xi = \left( {{\Omega } + \frac{1}{\gamma }E_{n} } \right)^{ - 1} \left( {Y - 1_{n} B} \right) $$

Here, $K\left({X}_{i},{X}_{j}\right)$ represents the kernel function, which is calculated as follows:

9 $$K\left({X}_{i},{X}_{j}\right)={\varphi \left({X}_{i}\right)}^{T}\varphi \left({X}_{j}\right)$$

The estimation of LSSVM regression can be achieved by solving the linear equations, as depicted in Eq. (10).

10 $$ f\left( X \right) = \mathop \sum \limits_{i = 1}^{n} \xi_{i} K\left( {X,X_{i} } \right) + {\text{B}} $$

In this study, the kernel function utilized is the radial basis function (RBF), a common choice in machine learning. The RBF maps the input sample points into a new eigenvector and calculates the point multiplication. Essentially, it transforms each sample point into an infinite-dimensional feature space^22^. The RBF can be computed using Eq. (11).

11 $$ K\left( {X,X_{i} } \right) = {\text{exp}}\left( { - \left\| {X - X_{i} } \right\|^{2} /\eta^{2} } \right) $$

In Eq. (11), $\eta$ signifies the kernel function parameter.

### Introduction to relational database

#### Meaning of relational database

A relational database is designed to work with relational models and stands as one of the most vital and prevalent DBMSs in use today. The market is dominated by relational databases, including well-known names like Sybase, Oracle, Informix, and Structured Query Language (SQL)^23^.

#### Advantages and disadvantages of relational database

**Figure 4:**
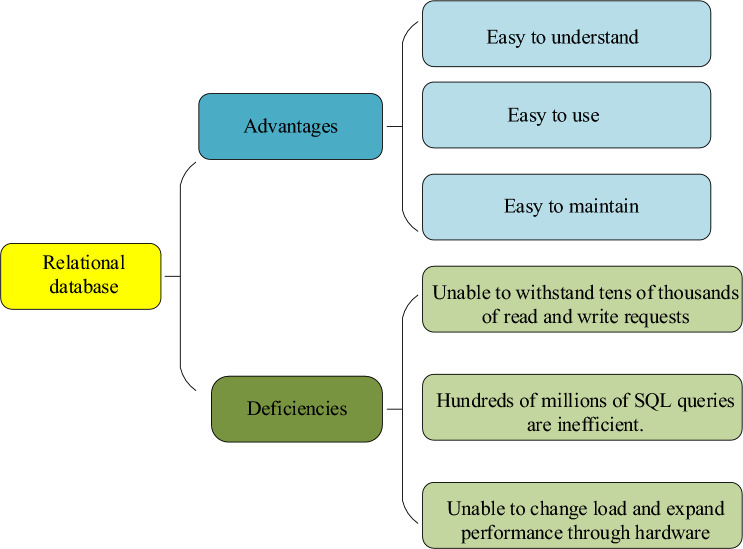
Advantages and disadvantages of relational database.

Figure 4 details the benefits and drawbacks of relational databases in comparison to other types of databases.

As depicted in Fig. 4, relational databases are known for their user-friendliness and ease of maintenance. Their structure closely aligns with the logical world, making them more comprehensible when compared to network structures. The use of SQL language enhances their ease of use and maintenance as it upholds entity integrity and user-defined data, reducing the risk of data inconsistencies^24^. However, one drawback is their limited capacity to handle high levels of concurrent read and write operations. When faced with tens of thousands of concurrent requests or hundreds of millions of SQL queries, relational databases can become inefficient. Furthermore, these databases face limitations in expanding performance and load capacity through hardware and service node modifications^25^.

### Construction of relational database load ECM

#### Theoretical foundation and model design

Within a relational database, users interact with the database using SQL statements. The actions performed by upper-level application programs on the database are translated into these SQL statements, and their execution significantly impacts the energy consumption of the database. The query optimizer generates a series of query plans to execute a submitted SQL statement. Consequently, an ECM is established for each SQL statement to ensure efficient performance while conserving energy^26^. The execution of SQL statements involves the consumption of the system’s Central Processing Unit (CPU), Random Access Memory (RAM), and hard disk resources. The cumulative energy consumption of these components constitutes the overall energy expenditure associated with SQL statements^27^.

The energy collection principle of self-powered WVSs and the LSSVM energy prediction algorithm are employed to address the ECM of the relational database^28^. In the initial stage, the energy consumption of the CPU is determined. The baseline power consumption of the database is established by configuring the power consumption for every 10,000 CPU instructions as $Ins \cdot watt$. The dynamic CPU power consumption is calculated using Eq. (12).

12 $$ P_{cpu} = Ins*Ins \cdot watt = Ins*\left( {10000/ Wins } \right) $$

In Eq. (12), $Ins$ signifies the count of CPU instructions, and ${\text{Wins}}$ represents the command power capability.

Next, the energy consumption of the hard disk is evaluated. Assuming the power consumed by a single block read operation is $Sin\cdot \text{ watt}$, the dynamic power consumption of the hard disk is computed by Eq. (13) when the disk IO type corresponds to a single block read.

13 $$ P_{disk} = Sin \cdot total*Sin \cdot watt $$

In Eq. (13), $Sin \cdot Total$ signifies the total number of single block read operations.

Assuming the power consumed by a multi-block read operation is $Multi \cdot watt$, the dynamic power consumption of the hard disk, when the disk IO type corresponds to multi-block read, is determined using Eq. (14).

14 $$ P_{disk} = Multi \cdot total *Multi \cdot watt $$

In Eq. (14), $Multi \cdot total$ represents the total count of multi-block read operations.

Now, when the SQL statement’s IO type is single block read, the system’s dynamic power during the execution of the SQL statement can be calculated as Eq. (15).

15 $$ Active \cdot power = Ins*Ins \cdot watt + Sin \cdot total*Sin \cdot watt $$

When the SQL statement’s IO type is multi-block read, the dynamic power consumed during the execution of the SQL statement is given by:

16 $$ Active \cdot power = Ins*Ins \cdot watt + Multi \cdot total*Multi \cdot watt $$

Furthermore, the dynamic energy consumption incurred during the execution of SQL statements in a mixed IO type environment can be determined by Eq. (17).

17 $$ \begin{aligned} Active \cdot energy & = Ins\left( {Ins \cdot watt* Ins \cdot time} \right) + Multi \cdot total\left( {Multi \cdot watt* Multi \cdot time} \right) \\ & \quad + Sin \cdot total\left( {Sin \cdot watt* Sin \cdot time} \right) \\ \end{aligned} $$

In Eq. (17), $Ins \cdot time$ represents the time required for the CPU to execute 10,000 instructions; $Multi \cdot time$ corresponds to the time needed for the disk to perform a multi-block read operation; $Sin \cdot time$ the time taken by the disk to execute a single block read operation.

This study introduces a model for predicting the energy consumption of SQL statements in relational databases. It combines self-powered WVSs with an LSSVM energy forecasting algorithm. Compared to other models, this approach offers several integrated advantages. Firstly, it considers critical components during the execution of SQL statements, such as CPU, memory, and hard drive usage. This comprehensive assessment helps researchers and practitioners better understand the impact of SQL statements on database energy consumption, providing more precise guidance for optimizing database performance and energy management. Secondly, by incorporating self-powered WVSs and the LSSVM algorithm, this model can collect and analyze real-time energy consumption data from the database, enabling accurate predictions of future energy consumption. This combination leverages the sensors’ self-powered capability and the LSSVM algorithm’s modeling ability, providing a reliable foundation for energy consumption prediction. Additionally, the model accounts for the impact of different types of I/O operations on energy consumption. It distinguishes between single block reads and multiple block reads, improving the accuracy of energy consumption prediction. Furthermore, the model also includes CPU instruction counts and execution time overhead in energy consumption prediction, providing a more realistic reflection of the impact of SQL statements on CPU energy consumption. By considering these factors comprehensively, this model can accurately predict and assess the energy consumption of SQL statements in relational databases. It offers tailored energy management strategies for database administrators, facilitating performance optimization and energy conservation. However, it is important to note that this model requires further validation and optimization in practical applications. Depending on different database environments and workloads, adjustments to model parameters and algorithms may be necessary to achieve the best energy consumption prediction results. Additionally, addressing energy consumption analysis in large-scale and complex database systems involving more factors remains a challenge that requires further research and improvement. Details of the model’s parameter design are illustrated in Table 1.

**Table 1:** Model parameter set.

Parameter	Meaning
Ins	Total CPU instructions
Wins	Instruction power capability
Sin	Power consumption of a single data block read
SinTotal	Total occurrences of single data block read
Multi	Power consumption of multiple data block read
MultiTotal	Total occurrences of multiple data block read
ActivePower	Dynamic system power during SQL statement execution
ActiveEnergy	Dynamic system energy generated during SQL statement execution

**Figure 5:**
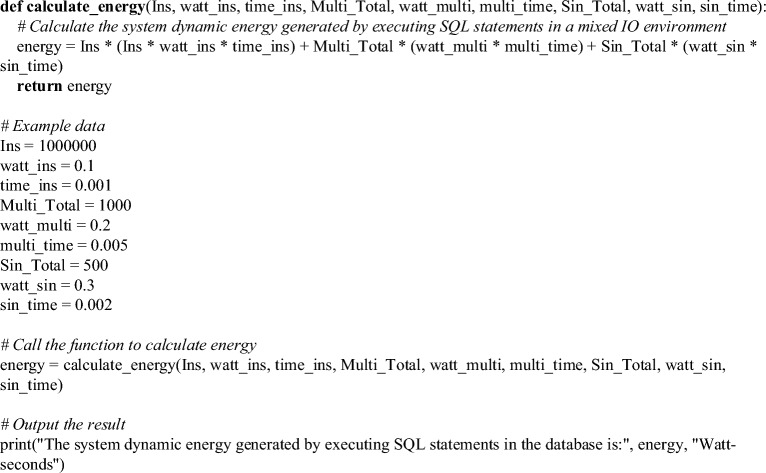
Data noise reduction process.

This study develops a predictive model for estimating the energy consumption of relational database workloads. Optimization steps are incorporated to enhance the model’s performance and address data noise issues during its application. Figure 5 represents the noise reduction optimization process.

As illustrated in Fig. 5, the process initiates with collecting and processing energy-related data, which can encompass variables such as CPU instruction counts, disk read/write operations, and power consumption. The study employs techniques like outlier removal, data smoothing, and normalization to refine the data and minimize inaccuracies. Subsequently, the paper selects a suitable energy consumption prediction model, a pivotal decision in this process. The LSSVM algorithm is employed to choose the model that best aligns with the specific data and target variables, ensuring a balance between prediction accuracy and computational efficiency. Next, computational formulas are translated into code, and the relevant data is utilized for energy consumption prediction. During this phase, verifying that input parameters align correctly with the computational formulas and are expressed in appropriate units is crucial. The code serves as a foundational framework, allowing the adjustment of input parameters and formulas to cater to specific prediction requirements. Finally, the prediction results are analyzed to assess the energy consumption generated during database execution. If the model’s prediction accuracy falls short of expectations, further adjustments and optimizations of model parameters or the consideration of a different model may be necessary. This iterative process continually refines and improves the accuracy and reliability of energy consumption prediction. In summary, energy consumption prediction through computational code is a multifaceted endeavor encompassing data collection and processing, model selection, formula implementation, result evaluation, and optimization. A systematic execution of these steps enhances the ability to effectively predict and manage energy consumption during database operations.

#### Description of model experiment environment

The experimental environment for the model is established based on pertinent literature and research findings, with the relevant parameters outlined in Table 2^29^.

**Table 2:** Experimental environment setting.

Item	Setting and model
Operating system	Windows Server 64 bit
CPU type	Intel Core E3-1225, @ 3.2 GHz, Quad Core
RAM	8 GB Haili Double Data Rate 31,600 MHz
Hard disk	Seagate ST1000DM004-1CH162,1 TB
DBMS	Oracle 11 g
Energy consumption measurement	Hopi power tester, USB smart version
Energy consumption data collection	Electric monitor data analysis system v1.0
Data acquisition frequency	1 Hz

#### Model implementation and energy consumption platform setup

**Figure 6:**
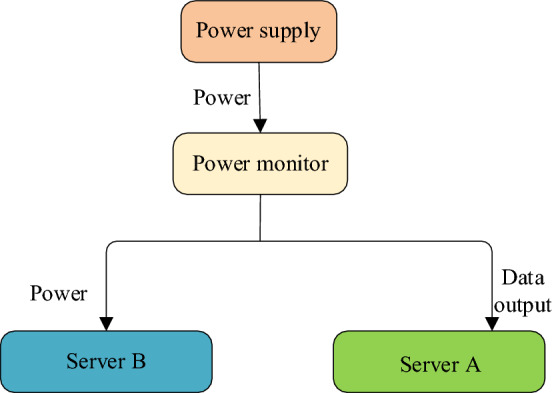
Energy consumption platform.

SQL statements serve as the standard interface for interfacing with and operating existing relational databases. Notably, a substantial portion of database resources, ranging from 70 to 90%, is dedicated to executing SQL statements. The execution time of SQL statements and their resource utilization significantly influence both the performance and energy consumption of the database load. Dual-computer communication is employed to ensure precise data collection of energy consumption. The Transaction Processing Performance Council-H (TPC-H) standard and a set of 22 complex SQL statements are adopted for experimental testing. The training set is based on a 10 GB database, while the test set employs a 1 GB database^30^. The architecture of the energy consumption prediction platform is depicted in Fig. 6.

As illustrated in Fig. 6, the ECM server, denoted as B, initiates sampling events to gather resource-related data. It is linked to power sources via a power meter. Server A is equipped with power monitoring software responsible for recording and scrutinizing energy consumption details. Additionally, it captures real-time data pertaining to current, voltage, and power through the power meter.

## Results and discussion

### Power source performance test of self-powered WVS

This study focuses on the evaluation of system performance under varying conditions, using energy consumption as a crucial metric. Energy consumption is a practical and relevant measure, directly impacting energy management and optimizing database systems. The model’s reliability and real-world applicability are assessed by examining energy consumption patterns and prediction accuracy.

Additionally, the research verified the self-sustaining capabilities of self-powered WVSs. Through experiments measuring voltage fluctuations during energy storage and consumption and analyzing energy variations under different operating modes, the study demonstrated that WVS nodes can efficiently accumulate energy and perform real-time tasks like network connectivity, command processing, and data transmission. This underscores the suitability of WVS nodes for applications in mechanical vibration testing, meeting their energy requirements. Furthermore, the study employs the tailored ECM designed for relational databases to predict and analyze the energy consumption associated with SQL statements. Through rigorous statistical analysis and training data, the ECM accurately predicts the energy consumption of SQL statements, a critical contribution to managing and optimizing energy in database queries. This achievement provides a foundational framework for the development of energy-efficient DBMSs. Lastly, the study delves into the examination of prediction errors under diverse environmental and resource conditions. The results underscore that in both isolated and competitive settings, the ECM exhibits lower prediction errors when compared to alternative models. Furthermore, the accuracy of ECM predictions varies under distinct memory resource conditions, highlighting the influence of environmental and resource factors on model performance. This recognition emphasizes the need for specific research and optimization targeting these unique conditions. In summary, this study conducts a comprehensive assessment of the model, utilizing multiple metrics to analyze the results and understand the contributing factors. These findings offer practical insights into energy management and database system optimization, providing guidance and a rationale for further research and refinements.

**Figure 7:**
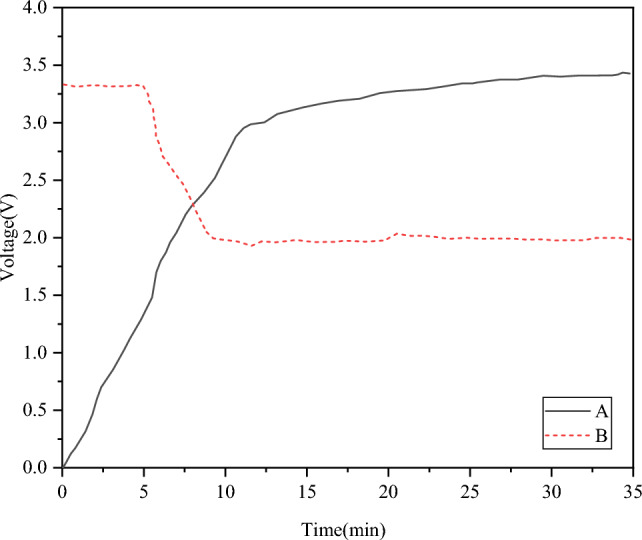
Voltage variation of energy storage mode and energy consumption mode.

Tests are conducted to examine the voltage fluctuations during the energy storage and energy consumption processes to validate the self-sustaining capabilities of self-powered WVSs. During the experiment, the speed regulator is adjusted to control the vibration test-bed’s speed, and the PET is fine-tuned to achieve a resonant state, resulting in a significant energy output. Figure 7 illustrates the voltage fluctuations observed during the energy storage and consumption modes.

In Fig. 7, A represents the curve for lightweight energy storage, and B represents the curve for energy consumption during networking. The self-powered WVS node has a total energy storage duration of 10 min, and each energy storage cycle increases the energy level from 0 to 3 V. Notably, a single energy storage cycle in the lightweight energy storage mode can sufficiently cover the energy consumption needed for multiple online activations of self-powered WVS nodes. With each energy storage cycle, these nodes can sustain network activities for up to 5 min. In summary, self-powered WVS nodes can rapidly accumulate energy, enabling real-time networking, command reception, and transmission, making them well-suited to meet the energy requirements of mechanical vibration testing applications.

### Prediction and analysis of SQL statement energy consumption

**Figure 8:**
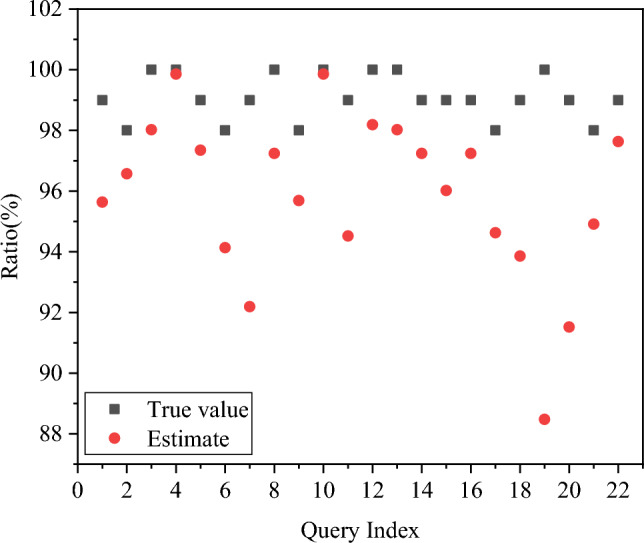
Predicted energy consumption of SQL statements.

This section aims to validate the accuracy of the proposed relational database-oriented ECM. The ECM undergoes training using the exclusive DBMS resources as provided by the training set. Subsequently, relevant statistical data are gathered to employ the proposed ECM to predict the energy consumption of 22 SQL statements. The specific results are presented in Fig. 8.

As depicted in Fig. 8, the ECM exhibits a reasonable capability to predict load energy consumption. The only exception is the 19th query statement, which has a prediction error exceeding 10%. In contrast, errors for the remaining queries remain below 10%. The average prediction error for load energy consumption is less than 6%, indicating a consistently low level of error. These results provide a foundational basis for the development of an energy-efficient green DBMS.

### Accuracy analysis of model prediction under different number of reading operations

#### Analysis of model prediction error rate under concurrent single-block and multi-block read operations

**Figure 9:**
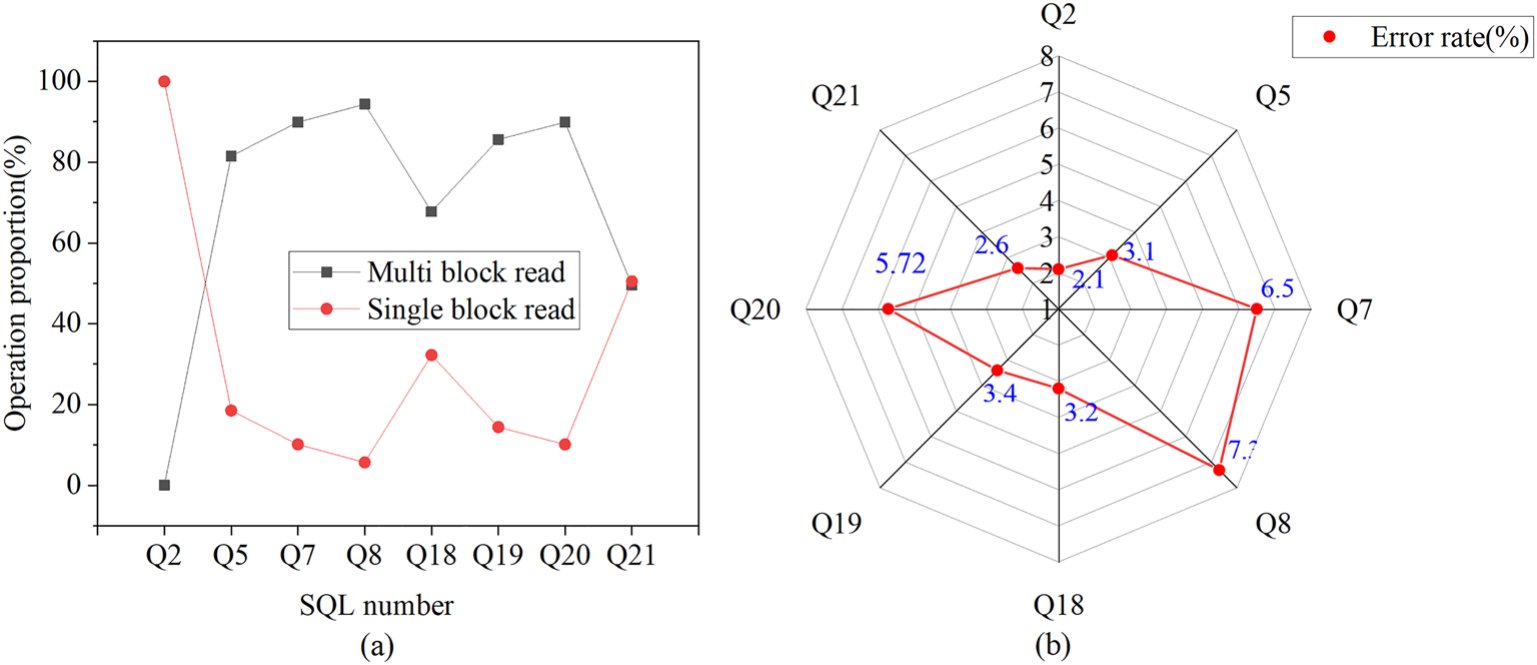
Analysis of operation proportion and model prediction error rate. (**a**) Proportion of multi-block and single-block read operations; (**b**) Comparison of model prediction error rates.

Eight of the 22 SQL statements used in the experiment are selected based on significant experimental results. These SQL statements are executed concurrently, involving both single-block and multi-block read operations. The analysis of operation proportions and model prediction error rates is presented in Fig. 9.

As shown in Fig. 9a,b, the operation ratio between multi-block and single-block read operations varies, and this difference influences the model’s prediction error rate. The minimum error rate is 2.1% when all operations are single-block reads. In contrast, Q8, which consists mainly of multi-block operations, exhibits the highest error rate. The accuracy of the proposed relational database-oriented ECM decreases as the proportion of multi-block reads increases relative to single-block reads.

#### Analysis of model prediction error rate under separate single-block reading and multi-block reading

**Figure 10:**
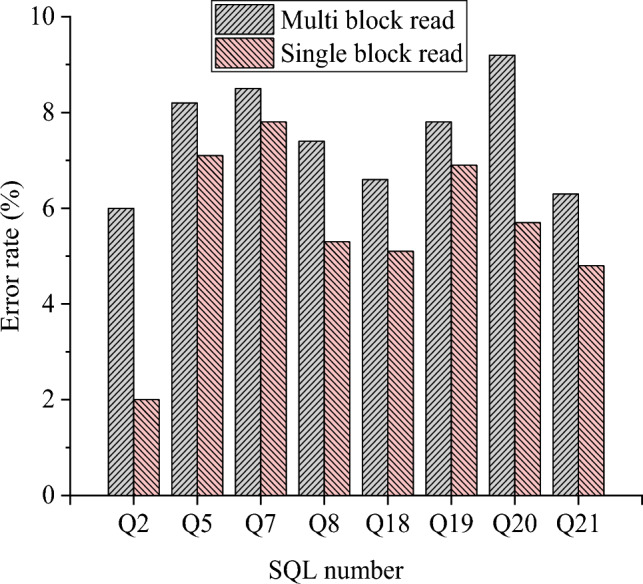
Error rate of the proposed ECM under single block reading and multi-block reading separately.

This section provides further verification of the relationship between multi-block and single-block read operations, and their respective influence on the accuracy of the proposed ECM is provided. The experimental results are presented in Fig. 10.

As illustrated in Fig. 10, the error rate for each SQL statement is relatively high during multi-block reads, while it is notably lower during single-block reads. When considering separate operations, the prediction accuracy of the proposed ECM is significantly higher during single-block reads compared to multi-block reads.

### Accuracy analysis of the proposed relational database-oriented ECM under different environments

#### Analysis of ECM error rates in different environments with sequential read operations

**Figure 11:**
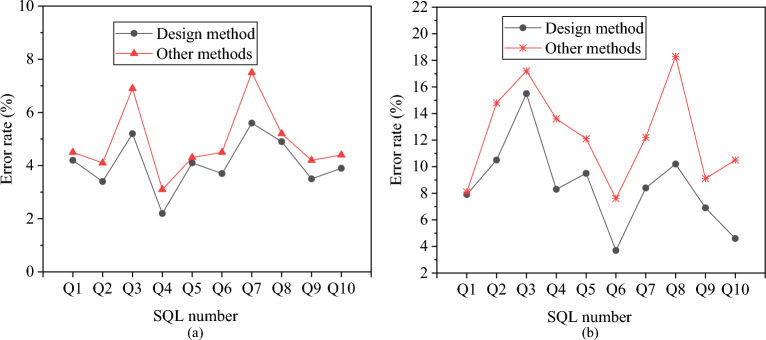
Prediction error rates for the proposed relational database-oriented ECM under sequential read operations, distinguishing between two settings: (**a**) error rates in an exclusive environment; (**b**) error rates in a competitive environment.

The error rate of the proposed ECM is analyzed under exclusive and competitive environments. An exclusive environment refers to a system where only the DBMS runs without other concurrent execution programs. In contrast, a competitive environment involves other programs in the system competing with the DBMS for resources. The proposed relational database-oriented ECM is compared with other models. It is important to note that these other models do not incorporate SVMs and are typical methods used for predicting database query energy consumption. The results for prediction error rates are presented in Fig. 11.

According to Fig. 11a, when the DBMS exclusively utilizes the system resources, the error rate of the proposed ECM is notably lower compared to other models. Furthermore, in a competitive environment, the error rate of the proposed relational database-oriented ECM remains lower. Consequently, the error rate for executing SQL statements is relatively low when using the proposed ECM in a DBMS exclusive environment.

#### Error rate analysis of proposed relational databased-oriented ECM under different environments based on random reading operation

**Figure 12:**
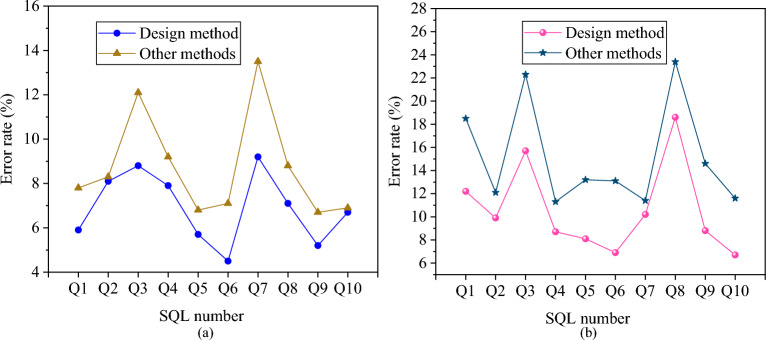
Prediction error rates for the proposed relational database-oriented ECM in two different environments. (**a**) The prediction error rate in an exclusive environment; (**b**) the prediction error rate in a competitive environment.

This section analyzes the error rates in exclusive and competitive environments by comparing the proposed ECM with other models under random reading operations. The results are presented in Fig. 12.

As depicted in Fig. 12, when DBMS exclusively utilizes the system resources, the error rates for each SQL statement exhibit significant variations. Except for Q2 and Q10, the proposed ECM demonstrates lower error rates for other SQL statements compared to alternative models. In a competitive environment, while the error rates for SQL statements are relatively high, the proposed ECM outperforms other models overall. The proposed relational database-oriented ECM maintains a lower error rate and higher accuracy during random reading operations.

### Accuracy analysis of the proposed relational database-oriented ECM under different memory resources

**Figure 13:**
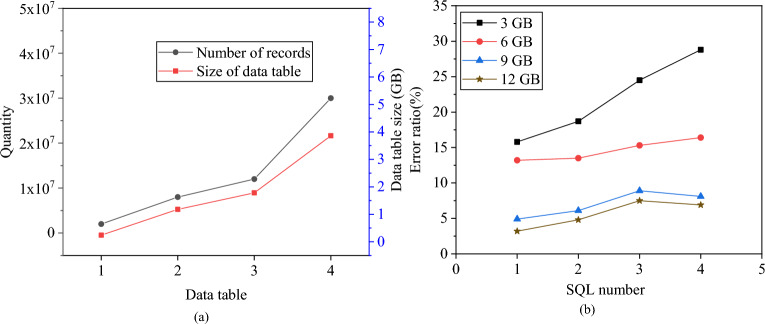
Number and size of data table records. (**a**) Number and size of data table records; (**b**) Error rate analysis of memory size on energy consumption prediction of the model.

In this section, various memory sizes (3 GB, 6 GB, 9 GB, and 12 GB) are configured to scan and sort four different data tables. The model’s prediction errors regarding energy consumption under different memory resources are computed. The specific results are presented in Fig. 13.

As depicted in Fig. 13, when the memory is set at 3 GB, the proposed ECM exhibits significant errors in read operations, with an average error exceeding 20%. This error rate tends to increase with larger SQL memory settings. However, when the memory is expanded to 9 GB and 12 GB, the average error decreases notably, revealing a clear correlation between the model’s error and memory capacity. The model’s error proportionally increases across the four memory settings mentioned as the data tables for SQL statements expand. This highlights a distinct relationship between the model’s error and the scale of SQL statement operations. The underlying reason is the persistent scarcity of memory resources. As the volume of temporary data generated by SQL statements rises, it leads to a subsequent increase in space operations and, consequently, higher prediction errors. Therefore, ensuring an ample supply of memory resources can enhance the accuracy and stability of the proposed ECM in read operations.

### Prediction and analysis of energy consumption

**Figure 14:**
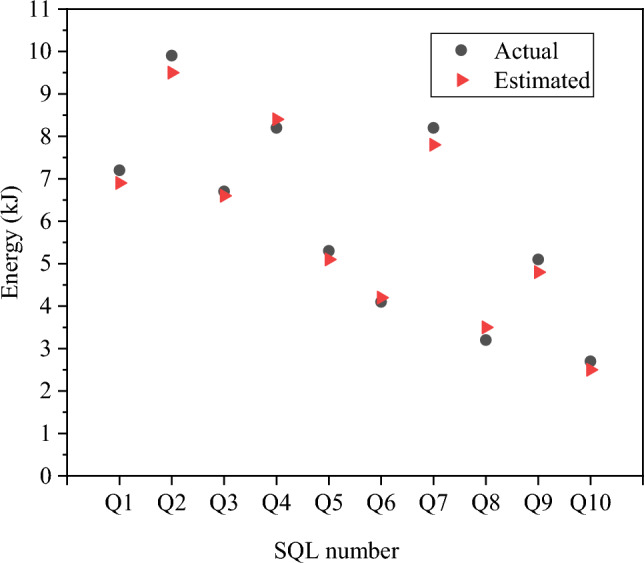
Comparison between predicted and real energy consumption.

In the following section, an analysis is conducted to compare the predicted energy consumption provided by the proposed relational database-oriented ECM against the actual energy consumption for ten distinct SQL query statements. The database size is set to 1 GB, and the results are presented in Fig. 14.

Evidently, the predicted energy consumption for query operations, as generated by the proposed relational database-oriented ECM, closely aligns with the actual values for various SQL statements, showcasing an error rate ranging from 1 to 4%. Except for specific SQL statements, the overall predicted values consistently fall below the actual values. This observation underscores the high level of accuracy exhibited by the proposed ECM in forecasting the load and energy consumption associated with relational databases.

### Discussion

This study empirically confirms the self-sustaining capabilities of self-powered WVS. The experiments clearly show that WVS nodes efficiently accumulated energy and executed real-time tasks like network connectivity, command processing, and data transmission. Even under conditions with minimal energy storage, a single cycle of energy accumulation can sufficiently power multiple active nodes, meeting the energy demands of mechanical vibration testing applications. As a result, WVS nodes are well-suited to fulfill the energy requirements of such testing applications.

Furthermore, the study introduces an ECM specifically designed for relational databases, enabling the prediction of energy consumption related to SQL statements. The experimental findings support the model’s practical utility by demonstrating its ability to reasonably predict energy consumption. For most SQL statements, prediction errors remain below 10%, with an average error rate of less than 6%. This model serves as a valuable tool and point of reference for the development of energy-efficient and eco-friendly DBMSs. In addition, the study assesses the model’s prediction accuracy under varying read operation scenarios. The results emphasize that the model excels in predicting single-block read operations but experienced a reduction in accuracy when dealing with a large volume of multi-block reads. This observation highlights the importance of considering different read operation scenarios during the design and optimization of the ECM. Furthermore, the study examines the model’s accuracy under different environmental conditions and memory resource availability. The experimental results reveal that when the DBMS monopolized system resources exclusively, the ECM exhibited lower prediction error rates. In competitive settings, the ECM still maintained relatively lower error rates. Moreover, ample memory resources positively impact ECM’s prediction accuracy and stability, particularly in read operations.

This study offers valuable guidance and insights for achieving energy conservation and environmentally friendly database management. While the model performs well, there is room for further optimization, particularly in scenarios involving a high number of multi-block reads, resource competition, and limited memory. Future research endeavors can concentrate on enhancing the model’s algorithms and techniques to improve its accuracy and stability, broadening its utility to a wider range of scenarios and applications.

## Conclusion

The study examines the architecture of self-powered WVS network nodes and the structure of vibration energy harvesting and management systems, which are critical components of energy-efficient and green database systems. Given the growing concerns regarding energy consumption and environmental impacts stemming from big data, researching the energy usage of relational database workloads assumes a pivotal role in advancing the development of energy-efficient green database systems. Drawing from the foundational principles of LSSVM, this study extends its scope to construct an energy consumption prediction model tailored for relational database workloads using LSSVM modeling techniques. The study establishes an energy consumption prediction platform and conducts experiments to evaluate the capabilities of self-powered WVSs. Moreover, the study assesses the model’s performance in predicting energy consumption related to SQL statements within relational databases. The study also examines the accuracy of the ECM under various conditions, including different numbers of read operations, environmental settings, and varying memory resource constraints. Finally, the study scrutinizes the model’s predictions related to ten distinct SQL query statements. The results are as follows:

1. Self-powered WVS nodes exhibit impressive energy accumulation capabilities. Within a mere 10 min, they can elevate stored energy from 0 to 3 V. In scenarios with lightweight energy storage, a single energy storage cycle proves sufficient to meet the energy requirements of multiple activations of self-powered nodes. Each energy storage cycle empowers nodes to sustain network connectivity and command transmission for up to 5 min. These findings underscore the ability of self-powered WVS network nodes to swiftly gather energy and perform real-time tasks, meeting strict time-related demands.
2. The energy consumption prediction model demonstrates effectiveness in forecasting workload energy consumption, maintaining prediction errors largely below 10%. The average error rate for energy consumption remains under 6%, signifying a low level of inaccuracies.
3. Prediction error rates within the model decrease proportionally with the increase in the ratio of multi-block reads to single-block reads. Accuracy witnesses a decline as the proportion of multi-block reads expands. When specifically considering single-block read operations, the model exhibits significantly higher accuracy compared to predictions for multi-block read operations.
4. In the context of sequential and random read modes, a comparison between prediction error rates in scenarios where the DBMS exclusively monopolizes resources and in competitive environments demonstrated relatively low prediction error rates for SQL statements in DBMS-exclusive conditions. Moreover, the prediction error rate for sequential read mode is even lower, signifying higher accuracy relative to other models.
5. As the data table size for SQL statement operations increases, the model’s prediction errors correspondingly escalate. This variation highlights a clear association between model inaccuracies and the volume of data involved in SQL statement operations.
6. A comprehensive analysis of the energy consumption predictions in comparison to actual values for ten distinct SQL query statements underscores the design model’s remarkable accuracy in forecasting the energy consumption of relational database workloads.

Nonetheless, it’s essential to acknowledge the limitations of this study. Firstly, the experimental setup did not completely replicate real-world situations, potentially influencing the reliability of our findings. Secondly, the sample size was relatively modest, which could affect the generalizability of the results. In future research, a more extensive investigation could encompass diverse types of databases and database workloads in various business contexts, thereby fine-tuning the model for improved prediction accuracy and reliability. Moreover, there is room for exploration into more advanced machine learning algorithms or deep learning frameworks, which could provide enhanced capabilities for analyzing and predicting database workload energy consumption.

### Supplementary Information

Supplementary Information.

## Data Availability

The data used to support the findings of this study are included within the article.
